# Dynamic gut microbiota changes in patients with advanced malignancies experiencing secondary resistance to immune checkpoint inhibitors and immune-related adverse events

**DOI:** 10.3389/fonc.2023.1144534

**Published:** 2023-04-11

**Authors:** Yanlin Zeng, Qingya Shi, Xinyu Liu, Hao Tang, Bo Lu, Qingyang Zhou, Yan Xu, Minjiang Chen, Jing Zhao, Yue Li, Jiaming Qian, Mengzhao Wang, Bei Tan

**Affiliations:** ^1^ Department of Gastroenterology, Peking Union Medical College Hospital, Peking Union Medical College & Chinese Academy of Medical Science, Beijing, China; ^2^ School of Medicine, Tsinghua University, Beijing, China; ^3^ Peking Union Medical College, Peking Union Medical College & Chinese Academy of Medical Science, Beijing, China; ^4^ Department of Internal Medicine, Peking Union Medical College Hospital, Peking Union Medical College & Chinese Academy of Medical Science, Beijing, China; ^5^ Department of Respiratory and Critical Care Medicine, Peking Union Medical College Hospital, Peking Union Medical College & Chinese Academy of Medical Science, Beijing, China

**Keywords:** gut microbiota, lung cancer, immune checkpoint inhibitor, immune-related adverse event, secondary resistance

## Abstract

**Background:**

Immune checkpoint inhibitors (ICIs) have been a breakthrough in cancer immunotherapy, but secondary resistance (SR) and immune-related adverse events (irAEs) are significant clinical dilemmas. Although the gut microbiota is associated with ICI efficacy and irAEs, the knowledge of longitudinal gut microbiota dynamics during SR and irAE development is still quite limited.

**Methods:**

This was a prospective observational cohort study of cancer patients initially receiving anti-programmed cell death-1 (PD-1) treatment between May 2020 and October 2022. Clinical information was collected to evaluate therapy response and AEs. Patients were divided into a secondary resistance (SR) group, a non-secondary resistance (NSR) group, and an irAE group. Fecal samples were longitudinally obtained from baseline across multiple timepoints and analyzed with 16S rRNA sequencing.

**Results:**

Thirty-five patients were enrolled, and 29 were evaluable. After a median follow-up of 13.3 months, NSR patients had a favorable progression-free survival (PFS) compared with SR (457.9 IQR 241.0-674.0 days vs. 141.2 IQR 116.9-165.4 days, *P*=0.003) and irAE patients (457.9 IQR 241.0-674.0 days vs. 269.9, IQR 103.2-436.5 days, *P*=0.053). There were no significant differences in the microbiota between groups at baseline. Several previously reported beneficial microbiomes for ICI efficacy including *Lachnospiraceae*, *Ruminococcaceae*, *Agathobacter*, and *Faecalibacterium* showed decreasing trends as secondary resistance developed, yet not achieved significance (*P*>0.05). Significant changes in butyrate-producing bacteria were also presented in the SR cohort (*P*=0.043) with a decreasing trend upon secondary resistance occurrence (*P*=0.078). While the abundance of IgA-coated bacteria was stable in the SR cohort, there was a temporary decrease upon ICI treatment initiation and reestablishment after continuation of ICI treatment in the NSR cohort (primary ICI response: 0.06, IQR 0.04-0.10; durable ICI response: 0.11, IQR 0.07-0.14; *P*=0.042). *Bacteroides* contributed most to the difference between baseline and irAE occurrence, which decreased after irAE occurrence (Baseline: 0.10 IQR 0.07-0.36; irAE occurrence: 0.08 IQR 0.06-0.12) and was restored upon irAE remission to a comparable level as baseline (irAE remission: 0.10 IQR 0.09-0.18).

**Conclusions:**

The development of SR and irAEs is related to the longitudinal dynamics of the intestinal microbiota. The investigation into the preventative and protective effects of enteric microbe manipulation strategies is further required.

## 1 Introduction

Immune checkpoint inhibitors (ICIs) have undoubtedly been a breakthrough in cancer therapy. Through blockade of cytotoxic T lymphocyte-associated antigen-4 (CLTA-4) or programmed death-1 (PD-1) pathways, ICIs recover exhausted cytotoxic T cells to restore anti-tumor immunity. ICIs have now been approved for many cancer types as first-line options (1, 2). Nevertheless, the therapeutic benefits of ICIs are limited by secondary resistance (SR) and immune-related adverse events (irAEs), both of which are common (3, 4).

Secondary resistance refers to the clinical scenario in which tumors initially respond to treatment but later progress without treatment discontinuation. It is generally accepted that ICIs significantly improve clinical outcomes, and while the initial response might last several years, responses are sustained in only a small proportion of patients (5–7). Nonetheless, knowledge about the mechanisms underlying secondary resistance is still quite limited (8–10), and there is a need for predictive and protective biomarkers, especially those based on manageable tumor-extrinsic features. On the other hand, as an undesirable consequence of immune reactivation, irAEs in various organs have been reported with different levels of severity, from mild rashes to life-threatening myocarditis. These often result in temporary or permanent discontinuation of ICIs (11).

The intestinal microbiota is a manageable factor related to ICI efficacy and AEs. Indeed, patients treated with antibiotics have been shown to have worse outcomes from ICI treatment, and fecal microbiota transplantation (FMT) from responders has been shown to enhance the antitumor effects of anti-PD-1 therapy in both murine models and patients (7, 12, 13). With respect to irAEs, supplementation with probiotics reduces colitis in mice. FMT has also successfully treated refractory ICI-related colitis, revealing the potential benefits of manipulating the gut microbiota to minimize irAEs (14, 15).

Although there is now a well-established association between the intestinal microbiota and ICI therapy, there is relatively little research on the longitudinal microbiota changes occurring during the development of secondary resistance and irAEs. Hence, we hypothesized that secondary resistance and irAEs are accompanied by changes in microbiota composition and diversity. We performed a longitudinal study of patients receiving anti-PD-1 treatment and explored the dynamic microbiota changes occurring in patients developing secondary resistance and irAEs.

## 2 Methods

### 2.1 Participants, inclusion, and exclusion criteria

**Figure 1:**
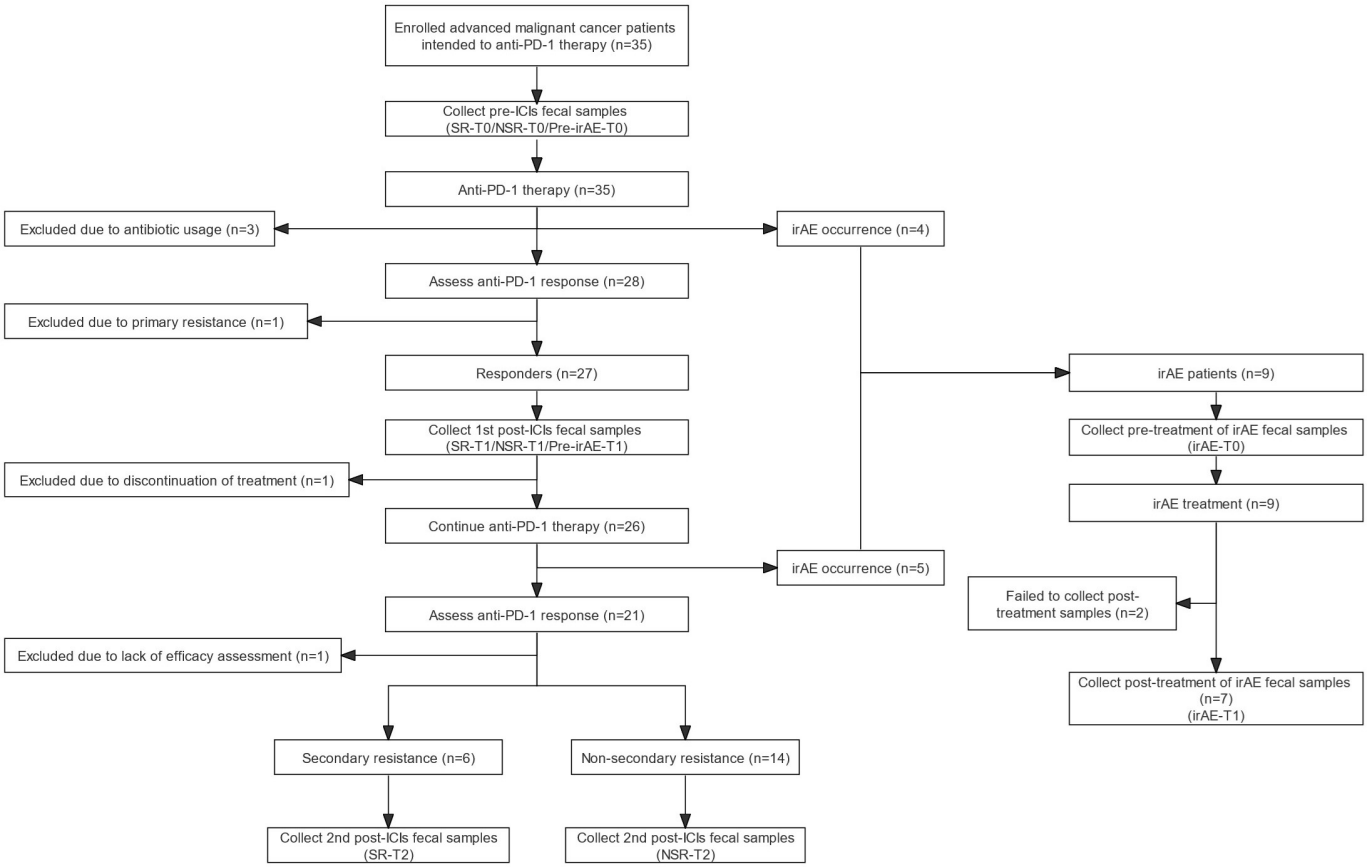
Flow diagram of participants and fecal sample collection. Flow diagram of enrollment and follow-up of patients receiving anti-PD-1/PD-L1 therapy. anti-PD-1, anti-programmed death-1; ICI, immune checkpoint inhibitor; irAE, immune-related adverse event; irAE-T_0_, irAE patients - after irAE occurrence; irAE-T_1_, irAE patients - after irAE remission; NSR, non-secondary resistance; NSR-T_0_, non-secondary resistance patients - before ICI treatment; NSR-T_1_, non-secondary resistance patients - primary response after ICI treatment; NSR-T_2_, non-secondary resistance patients - durable response after ICI treatment; PD-L1, programmed death-1 ligand; Pre-irAE-T_0_, irAE patients - baseline before ICI treatment; Pre-irAE-T_1_, irAE patients - after ICI treatment without irAE occurrence; SR, secondary resistance; SR-T_0_, secondary resistance patients - before ICI treatment; SR-T_1_, secondary resistance patients - primary response after ICI treatment; SR-T_2_, secondary resistance patients - secondary resistance after ICI treatment.

This observational longitudinal cohort study was conducted from May 2020 to October 2022 at Peking Union Medical College Hospital, Beijing, China. Patients with the following inclusion criteria were eligible for enrolment: (i) 18-80 years old; (ii) histologically diagnosed advanced thoracic cancer; (iii) initially treated with anti-PD-1 therapy; (iv) completed ≥ 4 cycles of ICI treatment or irAE occurrence; (v) completed ≥ 2 ICI efficacy evaluations; (vi) with or without concomitant usage of general anti-infective medication including proton pump inhibitors (PPIs), cardiovascular drugs, chemotherapy and antiemetics. Patients meeting any of following criteria were excluded: (i) primary resistance to ICI treatment; (ii) exposed to antibiotics and/or probiotics within 4 weeks before enrollment; (iii) requiring antibiotics and/or probiotics during follow-up. Patients were recruited from May 2020 to April 2021 and then followed up until data collection ended in October 2022. Out of the 35 patients enrolled, 27 patients established initial response to ICI and were followed up for long-term efficacy evaluation. According to subsequent ICI efficacy, they were divided into secondary resistance (SR) cohort and non-secondary resistance (NSR) cohort. Patients encountered irAEs were adjusted to the irAE group, regardless of ICI efficacy (**Figure 1**).

Demographic and clinical information were collected including gender, age, tumor type, disease stage, combined therapy, progression-free survival (PFS), overall survival (OS), treatment response, course of anti-PD-1 treatment, irAE type, and irAE grade. Response was assessed according to RECIST version 1.1 criteria (16). IrAEs were diagnosed according to National Comprehensive Cancer Network (NCCN) guidelines on ICI-related toxicities without restrictions in involved organs (11).

The Ethics Committee of Peking Union Medical College Hospital approved the study (ZS-3037). Written informed consent was obtained from all participants. This clinical study was registered on the Chinese Clinical Trial Registry (ChiCTR2200064816).

### 2.2 Sample collection and intestinal microbiota analysis

Fresh fecal samples were prospectively collected at multiple time points and stored in the Clinical Biobank, Medical Research Center, Peking Union Medical College Hospital, Chinese Academy of Medical Sciences. Samples from the SR cohort included SR-T_0_ (baseline before ICI treatment), SR-T_1_ (primary response after ICI treatment), and SR-T_2_ (secondary resistance after ICI treatment). Samples from the NSR cohort included NSR-T_0_ (baseline before ICI treatment), NSR-T_1_ (primary response after ICI treatment), and NSR-T_2_ (durable response after ICI treatment). Samples from the irAE cohort included Pre-irAE-T_0_ (baseline before ICI treatment, Pre-irAE-T_1_ (no irAE occurrence after ICI treatment), irAE-T_0_ (irAE occurrence after ICI treatment), and irAE-T_1_ (irAE remission after treatment).

Microbiota of fecal samples were detected by 16S rRNA gene V3-V4 amplicon sequencing on the Illumina NovaSeq 6000 platform. Amplicon sequence variants (ASVs) with abundance less than 5 among all samples were removed. After species annotation based on Silva and data normalization, ASVs were prepared for downstream analysis. Venn diagrams were plotted to seek common and differential ASVs between groups. The α diversity was assessed using the Shannon index. Beta diversity was assessed using the Bray-Curtis distance, and principal coordinate analysis (PCoA) was performed using Adonis. Similarity percentage (SIMPER) analysis was performed to identify microbiomes contributing to differences between groups at the genus level. Microbiota composition was also compared according to the relative abundance of the top 10 microbiomes at the phylum and genus levels. Differences in genera were evaluated by linear discriminant analysis effect size (LEfSe) at the genus level with an LDA score threshold of 3.

### 2.3 Statistical analyses

A formal sample-size calculation was not carried out for this longitude observation pilot study. Statistical analyses were performed in GraphPad Prism 8, SPSS 26.0, and R v4.0.3. Categorical variables are expressed as numbers and frequencies. Continuous variables are expressed as means ± SD or medians and interquartile ranges (IQR), as appropriate. The normality of distributions was examined using the Shapiro-Wilk test and Q-Q plots. Comparisons between groups with normal distributions were performed using Student’s *t*-test or one-way ANOVA, and the Mann-Whitney U test or Kruskal-Wallis test was used for groups with non-normal distributions. Pairwise longitudinal comparisons were conducted by repeated measures one-way ANOVA, mixed effects models, or Friedman’s test. A two-sided *P* < 0.05 was considered statistically significant.

## 3 Results

### 3.1 Demographic and clinical characteristics of the study cohort

Thirty-five patients with advanced thoracic malignancies initially receiving anti-PD-1 therapy were enrolled and followed. Six patients were excluded due to antibiotic exposure, primary resistance, or insufficient ICI treatment courses or evaluations. Among the 20 patients with primary responses to ICIs and qualified subsequent efficacy evaluation, 6 patients developed secondary resistance (SR) and 14 patients maintained their responses (NSR). The irAE group consisted of 9 patients regardless of efficacy evaluation (**Figure 1**).

The baseline characteristics of three cohort groups were generally well balanced. There were no significant differences in respect of age, gender, tumor type, disease stage, and adoption of combined chemotherapy or targeted therapy. Most of the participants received anti-PD-1 agents (93.1%). There was no significant difference of concomitant chemotherapy rates among SR patients, NSR patients and irAE patients (*P*=0.517). The most common chemotherapy regimens were pemetrexed combined with carboplatin (51.7%) or paclitaxel combined with carboplatin (34.5%). Other concomitant medication includes antiemetics (100.0%), cardiovascular drugs (44.8%), PPIs (20.7%), osmotic laxatives (44.8%), etc. (**Supplementary Figure 1**; **Supplementary Table 1**). Notably, after a median follow-up of 13.3 months, the NSR group achieved a significantly longer PFS (457.9 days, IQR 241.0-674.0) than the SR group (141.2 days, IQR 116.9-165.4, *P*=0.003) and the irAE group (269.9 days, IQR 103.2-436.5, *P*=0.053). There was a similar but non-significant trend for OS (*P*=0.275), with NSR patients having a favorable OS (479.5 days, IQR 286.8-672.2) without lethal events, followed by irAE patients (386.6 days, IQR 218.5-554.6) and SR patients (345 days, IQR 153.2-536.8). The most common irAE was rash (4/9) followed by colitis (2/9), pneumonitis (2/9), and myositis (1/9), mainly grade 1-2 (7/9) (**Table 1**).

**Table 1:** Demographics and characteristics of the patients.

Characteristics	Patients with secondary resistance (SR) (N=6)	Patients without secondary resistance (NSR) (N=14)	irAE patients (N=9)	*P*-value
Age - median (IQR) - years	62.0 (57.0-68.3)	63.0 (60.8-72.3)	66.0 (62.0-74.0)	0.376
Male gender - no. (%)	4 (66.7%)	9 (64.3%)	8 (88.9%)	0.485
Tumor type - no. (%)				0.693
NSCLC				
Lung adenocarcinoma	4 (66.7%)	7 (50.0%)	5 (55.6%)	
Squamous cell lung cancer	1 (16.7%)	4 (28.6%)	3 (33.3%)	
Others	1 (16.7%)	2 (14.3%)	0 (0.0%)	
SCLC	0 (0.0%)	1 (7.1%)	1 (11.1%)	
Disease stage - no. (%)				0.279
Stage III	0 (0.0%)	5 (35.7%)	3 (33.3%)	
Stage IV	6 (100.0%)	9 (64.3%)	6 (66.7%)	
Combined chemotherapy or targeted therapy - no. (%)	6 (100.0%)	14 (100.0%)	8 (88.9%)	0.517
Progression-free survival - median (IQR) - days	141.2 (116.9-165.4)	457.9 (241.0-674.0)	269.9 (103.2-436.5)	0.003
Overall survival - median (IQR) - days	345.0 (153.2-536.8)	479.5 (286.8-672.2)	386.6 (218.5-554.6)	0.275
Course of secondary resistance/irAEs occurrence -median (IQR) - no.	6 (5.8-6.5)	—	2 (1-4)	—

ICI, immune checkpoint inhibitor; IQR, interquartile; irAE, immune-related adverse event; NSCLC, non-small cell lung cancer; SCLC, small cell lung cancer.

### 3.2 Baseline intestinal microbiota of patients with and without secondary resistance and irAEs

**Figure 2:**
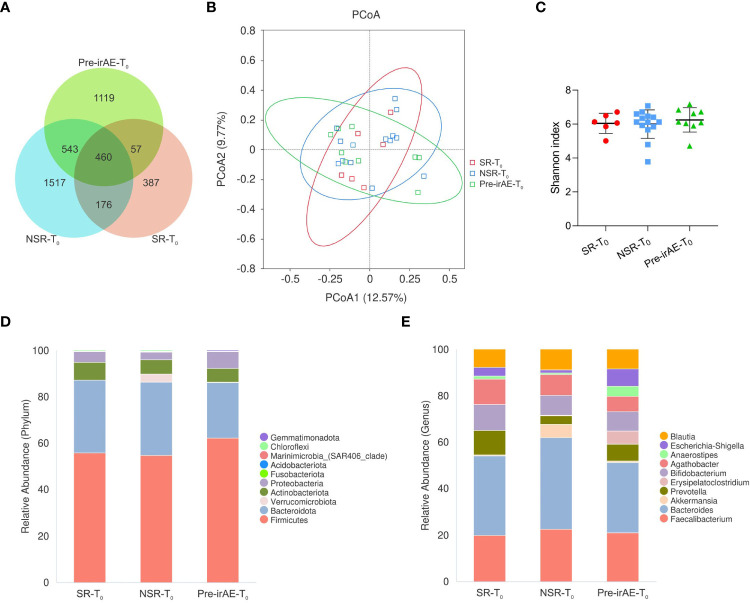
The baseline intestinal microbiota of patients with/without secondary resistance and irAEs. Fecal samples were collected at baseline from SR patients (SR-T_0_), NSR patients (NSR-T_0_), and irAE patients (Pre-irAE-T_0_) before ICI treatment. **(A)** Venn diagram of ASVs; **(B)** PCoA plot using the Bray-Curtis dissimilarity matrix; **(C)** α-diversity by Shannon index, with bars representing mean and standard deviation; **(D)** Histogram of intestinal microbiome relative abundance with the top 10 phyla. **(E)** Histogram of intestinal microbiome relative abundance with the top 10 genera. ASVs, amplicon sequence variants; ICI, immune checkpoint inhibitor; irAE, immune-related adverse event; NSR-T_0_, non-secondary resistance patients - before ICI treatment; PCoA, principal coordinates analysis; Pre-irAE-T_0_, irAE patients - before ICI treatment; SR-T_0_, secondary resistance patients - before ICI treatment.

We first compared the baseline microbiota of SR (SR-T_0_), NSR (NSR-T_0_), and irAE (Pre-irAE-T_0_) patients. There were 460 common ASVs and 387, 1537, 1119 distinctive ASVs in SR-T_0_, NSR-T_0_, and Pre-irAE-T_0_, respectively (**Figure 2A**). Although the SR-T_0_ had fewer differential ASVs compared with NSR-T_0_ and Pre-irAE-T_0_, the α-diversity by Shannon index was not significantly different (*P*=0.738) (**Figure 2C**). Also, the PCoA plot was not significantly different between the three patient groups (Adonis: *P*=0.821) (**Figure 2B**). *Verrucomicrobiota* and the corresponding genus *Akkermansia* were widely reported to benefit ICI efficacy (17, 18). The relative abundance of phylum *Verrucomicrobia* and genus *Akkermansia* are higher in NSR group compared to SR group and irAE group at baseline, according to the histograms of top 10 microbiota (**Figures 2D, E**). However, the relative abundances of phylum *Verrucomicrobia* (*P*=0.555) and genus *Akkermansia* (*P*=0.557) were low and failed to archieve significant difference. No significant dynamic changes were observed after SR, NSR or irAE occurred, either (**Supplementary Figure 4**; **Supplementary Table 2**).

### 3.3 Longitudinal intestinal microbiota of patients with and without secondary resistance

**Figure 3:**
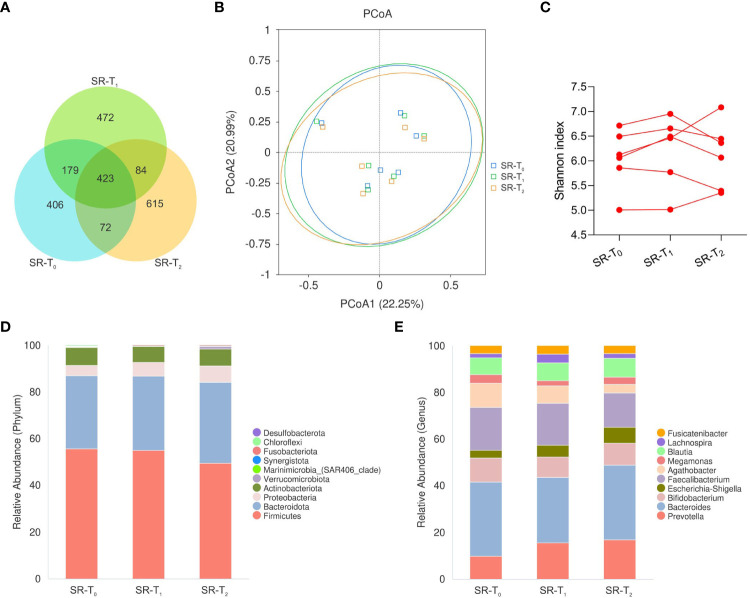
The longitudinal intestinal microbiota of patients with secondary resistance to ICIs. Fecal samples were sequentially collected from baseline (SR-T_0_) to primary response (SR-T_1_), to secondary resistance (SR-T_2_) in patients with secondary resistance. **(A)** Venn diagram of ASVs; **(B)** PCoA plot using Bray-Curtis distances; **(C)** Individual α-diversity by Shannon index; **(D)** Histogram of intestinal microbiota relative abundance with top 10 phyla; **(E)** Histogram of intestinal microbiota relative abundance with top 10 genera. ASVs, amplicon sequence variants; PCoA, principal coordinates analysis; SR-T_0_, secondary resistance patients - before ICI treatment; SR-T_1_, secondary resistance patients - primary response after ICI treatment; SR-T_2_, secondary resistance patients - secondary resistance after ICI treatment.

We first explored whether there were any dynamic changes in the intestinal microbiota in SR patients from baseline SR-T_0_ to primary response SR-T_1_ and secondary resistance SR-T_2_. There were 423 common ASVs, and 615 differential ASVs at SR-T_2_ compared with 406 at SR-T_0_ 406 and 472 at SR-T_1_ (**Figure 3A**). However, neither the α-diversity by the Shannon index (*P*=0.520) nor the β-diversity by PCoA (Adonis: *P*=1) revealed significant differences. The histograms of the relative abundance of the top 10 microbiota showed a steady composition over time at the phylum and genus levels (**Figures 3B–E**).

We also looked for the presence of longitudinal changes in the intestinal microbiota in NSR patients from baseline NSR-T_0_ to primary response NSR-T_1_ and to durable response NSR-T_2_. There were 970 common ASVs, and 1148 distinctive differential ASVs at NSR-T_0_, 1087 at NSR-T_1_, and 1163 at NSR-T_2_ (**Supplementary Figure 3A**). As in SR patients, the PCoA plot (Adonis: *P*=0.997) and α-diversity by Shannon index (*P*=0.931) revealed no significant differences between timepoints, nor did the histograms of the top 10 microbiota at the phylum and genus levels (**Supplementary Figures 3B–E**).

**Figure 4:**
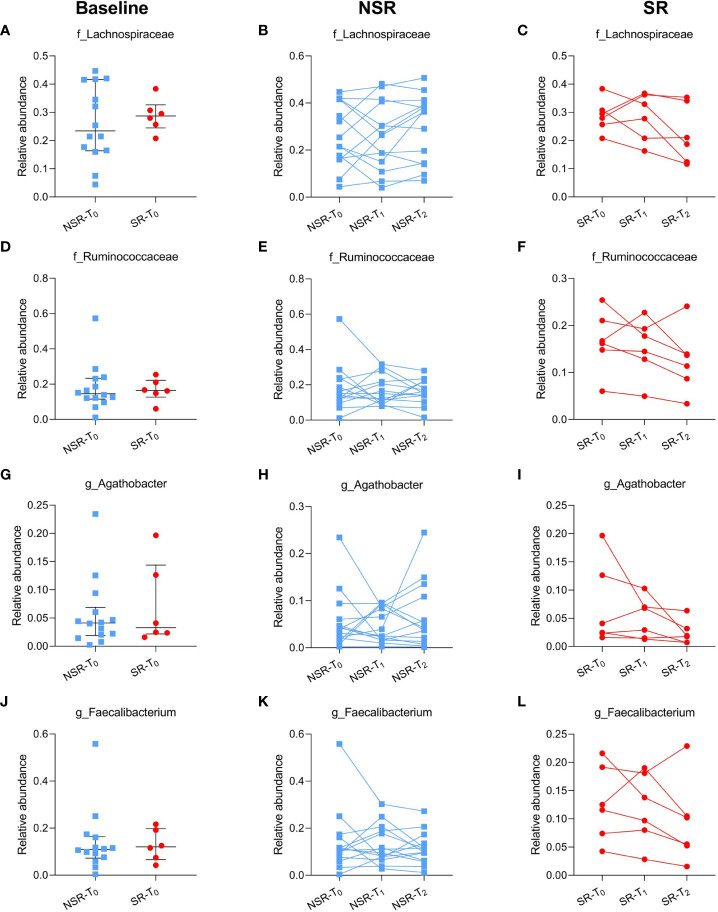
The relative abundance of reported microbiomes beneficial to ICI efficacy in patients with/without secondary resistance. The relative abundance of baseline **(A)** and individual changes in the *Lachnospiraceae* family in NSR patients **(B)** and SR patients **(C)**; baseline **(D)** and individual changes in the *Ruminococcaceae* family in NSR patients **(E)** and SR patients **(F)**; baseline **(G)** and individual changes in the *Agathobacter* genus in NSR patients **(H)** and SR patients **(I)**; baseline **(J)** and individual changes in the *Faecalibacterium* genus in NSR patients **(K)** and SR patients **(L)**. Medians and interquartile ranges are indicated. ICI, immune checkpoint inhibitor; NSR, non-secondary resistance; NSR-T_0_, non-secondary resistance patients - before ICI treatment; NSR-T_1_, non-secondary resistance patients - primary response after ICI treatment; NSR-T_2_, non-secondary resistance patients - durable response after ICI treatment; SR, secondary resistance; SR-T_0_, secondary resistance patients - before ICI treatment; SR-T_1_, secondary resistance patients - primary response after ICI treatment; SR-T_2_, secondary resistance patients - secondary resistance after ICI treatment.

Acknowledging that there was no notable difference in intestinal microbiota composition, we further compared the relative abundance of microbiomes reported to benefit ICI efficacy at baseline. First, the abundances of the *Lachnospiraceae* family (*P*=0.598), *Ruminococcaceae* family (*P*=0.810), *Agathobacter* genus (*P*=0.723), and *Faecalibacterium* genus (*P*=0.809) were comparable at baseline between SR and NSR patients. Interestingly, there were decreasing trends in the relative abundance of *Lachnospiraceae* (*P*=0.163), *Ruminococcaceae* (*P*=0.130), *Agathobacter* (*P*=0.142), and *Faecalibacterium* (*P*=0.226) as secondary resistance developed, although these were not significant. The relative abundance of the *Lachnospiraceae* family decreased from baseline (SR-T_0_: 0.29, IQR 0.26-0.30) and primary response (SR-T_1_: 0.30, IQR 0.23-0.35) to SR occurrence (SR-T_2_: 0.20, IQR 0.14-0.31). The abundance of *Ruminococcaceae* was relatively lower and also decreased upon development of secondary resistance (SR-T_0_: 0.16, IQR 0.15-0.20; SR-T_1_: 0.16, IQR 0.13-0.19; SR-T_2_: 0.13, IQR 0.09-0.14). A similar decreasing tendency was shown for *Agathobacter* and *Faecalibacterium*. In contrast, their relative abundances remained stable in NSR patients (*P*>0.05) (**Figure 4**; **Supplementary Table 2**).

**Figure 5:**
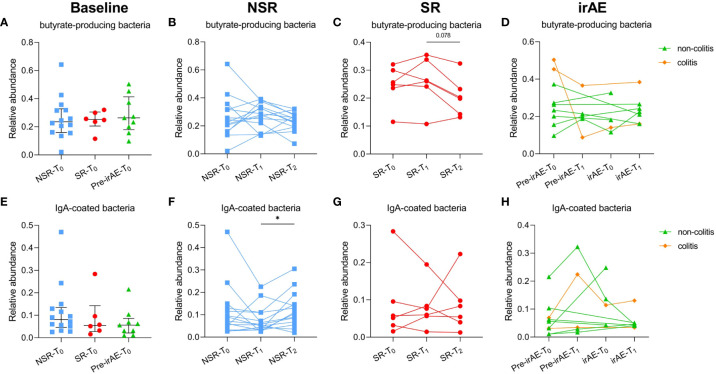
The relative abundance of butyrate-producing bacteria and IgA-coated bacteria in patients with/without secondary resistance and irAE occurrence. The relative abundance of baseline **(A)** and individual changes in butyrate-producing bacteria in NSR patients **(B)**, SR patients **(C)**, and irAE patients **(D)**; the relative abundance of baseline **(E)** and individual changes in IgA-coated bacteria in NSR patients **(F)**, SR patients **(G)**, and irAE patients **(H)**. Medians and interquartile ranges are indicated. irAE, immune-related adverse event; NSR, non-secondary resistance; NSR-T_0_, non-secondary resistance patients - before ICI treatment; NSR-T_1_, non-secondary resistance patients - primary response after ICI treatment; NSR-T_2_, non-secondary resistance patients - durable response after ICI treatment; SR, secondary resistance; SR-T_0_, secondary resistance patients - before ICI treatment; SR-T_1_, secondary resistance patients - primary response after ICI treatment; SR-T_2_, secondary resistance patients - secondary resistance after ICI treatment.

Based on the above results, individual beneficial bacteria may have only a limited effect on ICI efficacy. Given the complexity of microbiota composition and host-microbiome interactions, we further explored the longitudinal changes of well-known functional microbiome clusters. Butyrate-producing bacteria included *Roseburia*, *Ruminoccocus*, and *Clostridium* (19) etc., while IgA-coated bacteria consisted of *Bifidobacterium* and *Streptococcus* (20) etc., and the baseline abundances of both butyrate-producing bacteria (*P*=0.796) and IgA-coated bacteria (*P*=0.640) were similar in SR and NSR patients (**Figures 5A, E**). Interestingly, there were significant changes in butyrate-producing bacteria as secondary resistance developed (*P*=0.043); there was a decreasing trend in relative abundance on occurrence of secondary resistance (SR-T_1_: 0.26, IQR 0.25-0.32; SR-T_2_: 0.20, IQR 0.16-0.22; *P*=0.078), which was not observed in the NSR cohort. While the abundance of IgA-coated bacteria was stable in the SR cohort, there was a decreasing trend upon treatment initiation in the NSR cohort that reestablished itself after several courses of ICI treatment (NSR-T_1_: 0.06, IQR 0.04-0.10; NSR-T_2_: 0.11, IQR 0.07-0.14; *P*=0.042) (**Figure 5**; **Supplementary Table 2**).

### 3.4 The longitudinal intestinal microbiota of patients with irAEs

**Figure 6:**
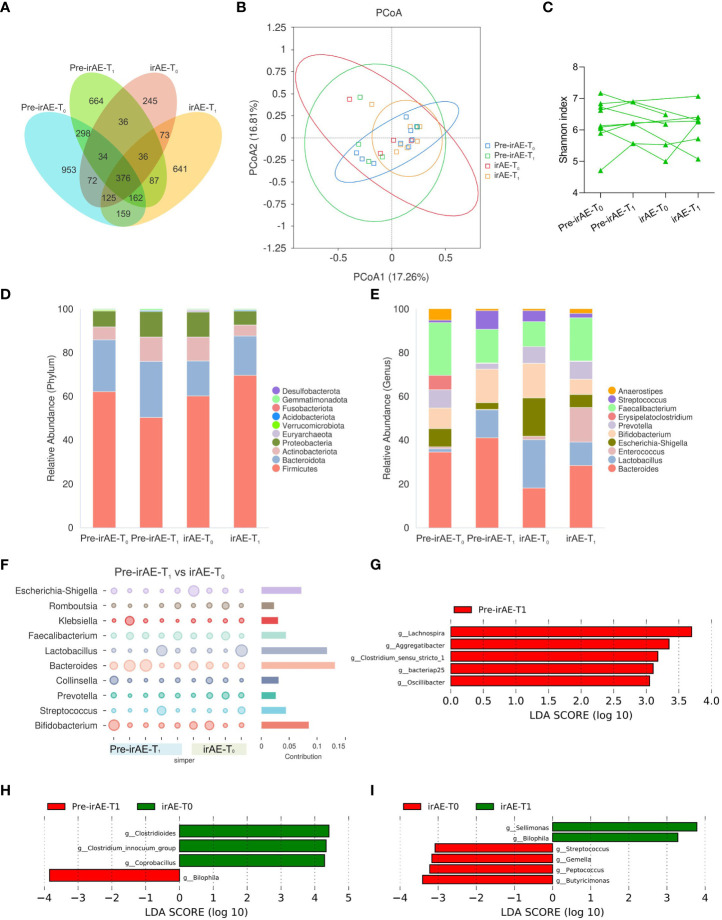
Longitudinal intestinal microbiota profiles in patients with irAEs. Fecal samples were sequentially collected from baseline Pre-irAE-T_0_ to Pre-irAE-T_1_ after ICI treatment without irAEs, irAE-T_0_ with irAE occurrence, and irAE-T_1_ with irAE remission. **(A)** Venn diagram of ASVs; **(B)** PCoA plot using Bray-Curtis distances; **(C)** Individual α-diversity by Shannon index; **(D)** Histogram of intestinal microbiota relative abundance with top 10 phyla; **(E)** Histogram of intestinal microbiota relative abundance with top 10 genera; **(F)** SIMPER analysis at genus level between Pre-irAE-T_1_ and irAE-T_0_. LEfSe analysis **(G)** between Pre-irAE-T_0_ and Pre-irAE-T_1_, **(H)** between Pre-irAE-T_1_ and irAE-T_0_; **(I)** between irAE-T_0_ and irAE-T_1_ at the genus level with threshold LDA score of 3. ASVs, amplicon sequence variants; irAE, immune-related adverse event; irAE-T_0_, irAE patients - after irAE occurrence; irAE-T_1_, irAE patients - after irAE remission; LEfSe, linear discriminant analysis effect size; PCoA, principal coordinates analysis; Pre-irAE-T_0_, irAE patients - baseline before ICIs treatment; Pre-irAE-T_1_, irAE patients - after ICI treatment without irAE occurrence; SIMPER, similarity percentage.

We next explored whether there were dynamic changes in the intestinal microbiota in patients who developed irAEs: from baseline Pre-irAE-T_0_ to Pre-irAE-T_1_ after ICI treatment without irAEs, irAE-T_0_ with irAE occurrence, and irAE-T_1_ with irAE remission. Upon irAE occurrence, there were fewer distinct differential ASVs at irAE-T_0_ (n=245) than at irAE-T_1_ (n=641), Pre-irAE-T_1_ (n=664), and Pre-irAE-T_0_ (n=953) (**Figure 6A**). The PCoA plot (Adonis: *P*=0.737) and Shannon index (*P*=0.297) did not reveal significant differences over time (**Figures 6B, C**).

To further identify if certain microbiomes were associated with the development of irAEs, SIMPER analysis was performed, which revealed that the *Bacteroides* genus contributed most to the differences between the Pre-irAE-T_1_ group and the irAE-T_0_ group (**Figure 6F**). Consistently, the relative abundance of the *Bacteroides* genus decreased after irAE occurrence (Pre-irAE-T_1_: 0.10, IQR 0.07-0.36; irAE-T_0_: 0.08, IQR 0.06-0.12). Upon irAE remission with glucocorticoid treatment, the abundance of the *Bacteroides* genus was restored to comparable levels as before irAE development (Pre-irAE-T_1_: 0.10, IQR 0.07-0.36; irAE-T_1_: 0.10, IQR 0.09-0.18) (**Figures 6D, E**; **Supplementary Table 2**). These differences persisted when the two patients with colitic irAEs were excluded from the analysis, excluding the possible influence of a microbiota disorder caused by colitis itself (**Supplementary Figure 5**). LEfSe analysis was performed to identify the main different genera between longitudinal groups. *Lachnospira* and *Aggregatibacter* were enriched after ICI treatment (Pre-irAE-T_1_), and the relative abundance of *Clostridiodes* increased while that of *Bilophila* decreased after irAE occurrence (irAE-T_0_). Of note, *Bilophila* increased after irAE remission (irAE-T_1_) (**Figures 6G–I**). However, both butyrate-producing and IgA-coated bacteria showed no significant differences over time (**Figures 5D, H**).

### 3.5 Impact of concomitant medications on intestinal microbiota

Since cardiovascular drugs were used in a long-term manner before anti-cancer therapy, the baseline microbiota could be sufficiently representative. The PCoA plot showed that there was no significant difference of baseline microbiota in patients with or without concomitant cardiovascular drugs (Adonis: *P*=0.136). As for PPIs and osmotic laxatives, which were mainly initially administrated along with anti-cancer therapy, we analyzed the dynamic changes of microbiota from baseline to post-PPIs or osmotic laxatives exposure. There was no significant longitudinal difference in microbiota before and after administration of either PPIs (Adonis: *P*=0.969) or osmotic laxatives (Adonis: *P*=0.996) (**Supplementary Figure 2**).

## 4 Discussion

Here we found that patients receiving anti-PD-1 treatment did not experience significant changes in intestinal microbiota diversity but some alterations in microbiota composition. SR patients had a generally stable microbiota composition with some specific changes in several beneficial microbes and functional clusters, while irAE patients experienced a shift in microbiota composition.

The SR and NSR cohorts had similar microbiota diversity and composition at baseline, which is reasonable since both achieved primary responses. In longitudinal analyses, both NSR and SR cohorts maintained stable intestinal microbiota diversity and general composition. The gut microbiota is a homeostatic system influenced by various factors such as the diet and environment, and indeed multiple rounds of FMT are generally needed to establish a new microbiota composition (12, 14). We found that anti-PD-1 treatment only had a minor impact on microbiota homeostasis in cohorts without irAEs. This suggests that the intestinal microbiota of each individual is relatively stable. However, we did found some differences among certain microbes reported to be associated with ICI efficacy. We examined specific changes in beneficial microbes and functional bacterial clusters. Baseline enrichment of microbes including *Agathobacter*, *Faecalibacterium*, *Ruminococcaceae*, and *Lachnospiraceae* have been positively correlated with anti-PD-1 inhibitor efficacy in NSCLC patients (21, 22). They were presented with similar abundance at baseline regardless of therapeutic response in our cohorts. However, in our longitudinal observations, there were decreasing trends in these beneficial microbes in SR but not NSR patients, which suggests a relationship between the development of secondary resistance and specific intestinal microbiota components.

With respect to functional clusters, we focused on butyrate-producing bacteria and IgA-coated bacteria. Fecal butyrate has been reported to be positively associated with response to not only chemotherapy but immunotherapy (23, 24). However, oral supplement of butyrate was not confirmed to promote immune response in mouse model (25). It is still unclear whether butyrate-producing bacteria play a role in ICIs efficacy. Hence, we analyzed the dynamic changes of fecal butyrate-producing bacteria. Remarkably, while the baseline relative abundance of butyrate-producing bacteria was comparable between SR and NSR patients, there was a significant decrease of the abundance after SR occurrence, but not in the NSR cohort. These supported the positive role of butyrate-producing bacteria in ICIs response. Also, it is worth noticing that the role of circulating short chain fatty acids in ICIs efficacy is still not clear. Nomura et al. showed that serum butyrate was slightly higher in responders to anti-PD-1 therapy than non-responder but without significance, while Coutzac et al. founded that circulating butyrate was inversely correlated with anti-CTLA-4 efficacy (24, 25). The circulating butyrate concentrations seems low and not parallel to the fecal counterparts in these studies, which may be due to the poor absorption into circulation, because butyrate serves as local nutrient for mucosal cells and gluconeogenic substrate for hepatic cells. The assessment of serum and fecal butyrate concentrations, as well as the potential mechanism exploration is further needed. In contrast, IgA-coated bacteria are a pro-inflammatory subset (20), and we wondered if their abundance would decrease with the development of secondary resistance and a suppressed immune response. Here we found that their abundance reduced after ICI initiation but later recovered in NSR cohorts, while stable abundance was observed in SR patients. It would be interesting to establish whether this temporary disturbance and subsequent maintenance of pro-inflammatory bacteria in NSR patients are predictive of sustained treatment responses.

In the irAE cohort, there were no overt changes in microbiota diversity but there were changes in compositional dynamics. There is a large body of evidence showing an association between microbiota composition and irAE occurrence. Certain microbes such as *Bifidobacterium* and *Bacteroides* are overrepresented in irAE patients compared with non-irAE patients (26). In this longitudinal study, the relative abundance of *Bacteroides* in irAE patients decreased from baseline to irAE occurrence and was partially restored upon irAE remission. Here we reinforced its negative association with irAE development. Moreover, we identified *Bilophila* as a differential biomarker before irAE occurrence and after irAE remission. *Bilophila* has been reported to be a pathobiont mediating the stimulative effect of bile acids on colitis in mouse experiments (27). The increased abundance of *Bilophila* also supports a link between an animal-based diet and outgrowth of microorganisms capable of boosting inflammatory bowel disease (IBD) in humans (28). Conversely, we found that *Bilophila* may protect against irAEs, with this discrepancy probably due to species differences or the specific and different mechanisms underlying irAEs and IBD. Of note, we performed the analysis again after removing the two participants with colitic irAE, and the results were fairly similar. These implied the difference of microbiota may play a role in irAE occurrence, but not resulted from microbiota disturbance by colitis itself. Moreover, we analyzed the abundance of functional bacterial subsets in irAE cohorts. Butyrate has been reported to protect against irAE colitis, but its role in other types of irAE remains elusive (7). IgA coating identified a colitogenic cluster affecting susceptibility to IBD, whereas its function in irAEs has yet to be established (20). However, the relative abundance of these two clusters were constant in our irAE cohorts.

Although we focused on relationship between immunotherapy and gut microbiota, indeed complex concomitant chemotherapy and other medications might also influence the microbiota (29). Most of our participants had ICIs combined with chemotherapy as standard first-line treatment, leading to difficulty in excluding the influence from chemotherapy. There was no difference of chemotherapy rates between SR, NSR and irAE groups, so it is unlikely that difference of microbiota among these groups resulted from biased chemotherapy. Future studies to self-compare the initial ICIs combined with chemotherapy treatment and the subsequent ICIs standalone maintenance will help address the longitudinal confounding bias of chemotherapy. Interestingly, it is reported that PPIs and osmotic laxatives in alimentary tract drugs and platelet aggregation inhibitors (e.g. aspirin) in antithrombotic drugs can exert effect on microbiota. Other cardiovascular drugs such as angiotensin II antagonists may also play a role. PPIs and osmotic laxatives were consistently associated the same species alterations regardless of duration of administration and dosage (30). From our preliminary analysis, cardiovascular drugs, PPIs and osmotic laxatives did not show significant influence on microbiota. Therefore, we preliminarily consider that the microbiota differences in our study were mainly owing to the application of ICIs.

Our study has several limitations, the most notable being the small sample size. We observed several trends in microbiome changes, which although interesting were not significant. As a prospective longitudinal cohort, it took time to observe the development of secondary resistance and irAE occurrence with timely fecal sampling during follow-up. Moreover, participants who continued visiting hospital might represent those with better efficacy outcomes. Most patients in our study experienced less severe irAEs, which may have represented a bias. We made efforts to avoid loss to follow-up, but nevertheless the bias and reduced power resulting from the small sample size is somehow hard to address. Further expanding the sample size and extending the observation time to verify our preliminary results in this pilot study is warranted. Second, we performed 16S rRNA gene V3-V4 amplicon sequencing, but metagenomics sequencing would be better suited to characterizing multiple species with host-microbiota interactions, metabolites, and relevant gene pathways. Third, our patients had immunotherapy with complex concomitant medications. Future study with more strict recruitment criteria or subgroup analysis is required to address the effect of ICIs itself. Lastly, pre-clinical models with interventions are required to establish causality. As a pilot study of small sample size, we should generalize the results with caution, but we still detected an association between dynamic changes in the gut microbiota and the effects of ICI, so we encourage further studies on microbiota-host interactions.

Secondary resistance is common and profoundly limits patient survival. Although research on drugs targeting novel immune checkpoints or VEGF signaling have shown some favorable effects in NSCLC patients with ICI resistance, effective clinical strategies to overcome secondary resistance are still limited (10). In addition to mechanisms such as mutational burden and altered tumor microenvironment (9, 31), secondary resistance is related to the intestinal microbiota. Enteric manipulation before or after secondary resistance might help establish the preventive and protective role of microbiota in this important clinical context. With respect to irAE treatment, several agents such as glucocorticoids and/or biologics can be used but it is still of clinical significance to establish the role of microbiota and its capacity to modify irAE occurrence to avoid immunotherapy discontinuation.

In conclusion, we found that patients with secondary resistance to ICI therapy had a decrease in beneficial bacteria including a butyrate-producing subset. IrAE patients experienced longitudinal changes in general microbiota composition, and *Bacteroides* and *Bilophila* were negatively associated with the development of irAEs. Further, larger investigations over longer timeframes are needed to consolidate this finding together with microbiota intervention studies.

## Data availability statement

The data presented in the study are openly available in Figshare at http://doi.org/10.6084/m9.figshare.21900462.

## Ethics statement

The studies involving human participants were reviewed and approved by The Ethics Committee of Peking Union Medical College Hospital (ZS-3037). The patients/participants provided their written informed consent to participate in this study.

## Author contributions

YZ and QS analyzed and interpreted data, drafted the manuscript. XL, HT, QZ analyzed microbiota data. LB, YX, MC, JZ, YL enrolled patients and collected samples. JQ, MW contributed to conception and design the study. BT designed and performed the study, critical revised the manuscript, supplied the funding support. All authors contributed to the article and approved the submitted version.
