# Supplementary material for: Dynamic gut microbiota changes in patients with advanced malignancies experiencing secondary resistance to immune checkpoint inhibitors and immune-related adverse events
Source: Front Oncol. 2023 Apr 11;13:1144534. doi: 10.3389/fonc.2023.1144534 (PMC10126279; doi:10.3389/fonc.2023.1144534)
Supplement: Supplementary file 1 [file DataSheet_1.docx]

Supplementary Material

Dynamic Gut Microbiota Changes in Patients with Advanced Malignancies Experiencing Secondary Resistance to Immune Checkpoint Inhibitors and Immune-Related Adverse Events

Yanlin Zeng†, Qingya Shi†, Xinyu Liu, Hao Tang, Bo Lu, Qingyang Zhou, Yan Xu, Min-jiang Chen, Jing Zhao, Yue Li, Jia-ming Qian, Meng-zhao Wang, Bei Tan^*^

*** Correspondence:** Corresponding Author: tanbei0626@aliyun.com

**Data availability**: The datasets presented in this study can be found in Figshare at http://doi.org/10.6084/m9.figshare.21900462.

**
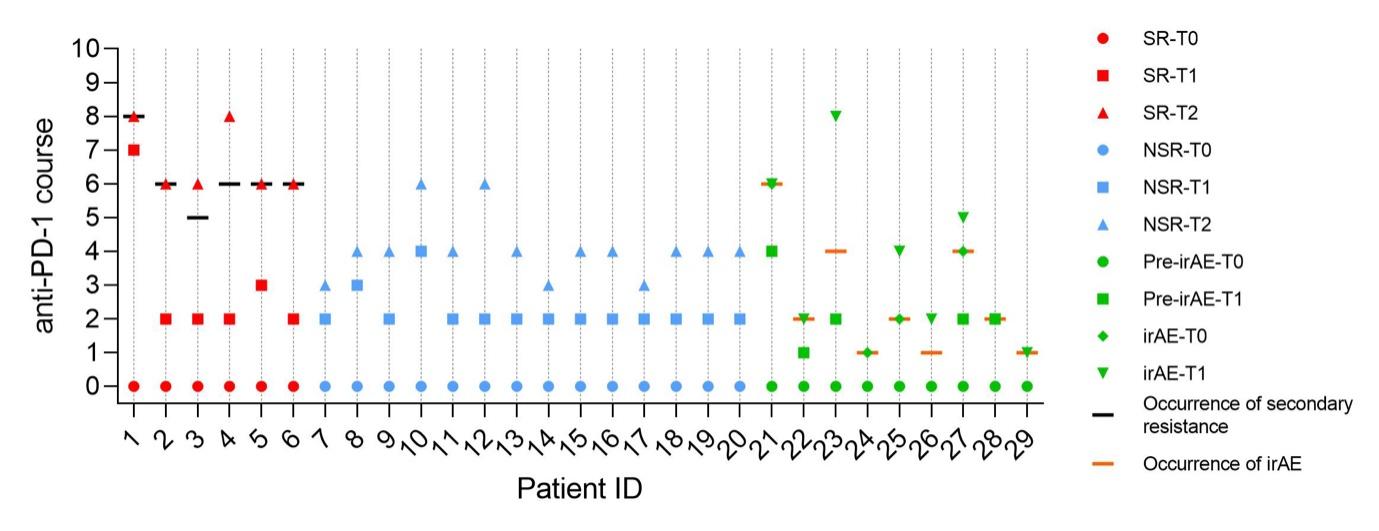
**

**Supplementary Figure 1. Number of immunotherapy courses.**

Number of anti-PD-1 courses at multiple timepoints of fecal sample collection, secondary resistance occurrence, and irAE occurrence.

Abbreviations: anti-PD-1, anti-programmed death-1; ICI, immune checkpoint inhibitor; irAE, immune-related adverse event; irAE-T_0_, irAE patients - after irAE occurrence; irAE-T_1_, irAE patients - after irAE remission; NSR, non-secondary resistance; NSR-T_0_, non-secondary resistance patients - before ICI treatment; NSR-T_1_, non-secondary resistance patients - primary response after ICI treatment; NSR-T_2_, non-secondary resistance patients - durable response after ICI treatment; Pre-irAE-T_0_, irAE patients - baseline before ICI treatment; Pre-irAE-T_1_, irAE patients - after ICI treatment without irAE occurrence; SR, secondary resistance; SR-T_0_, secondary resistance patients - before ICI treatment; SR-T_1_, secondary resistance patients - primary response after ICI treatment; SR-T_2_, secondary resistance patients - secondary resistance after ICI treatment.

**
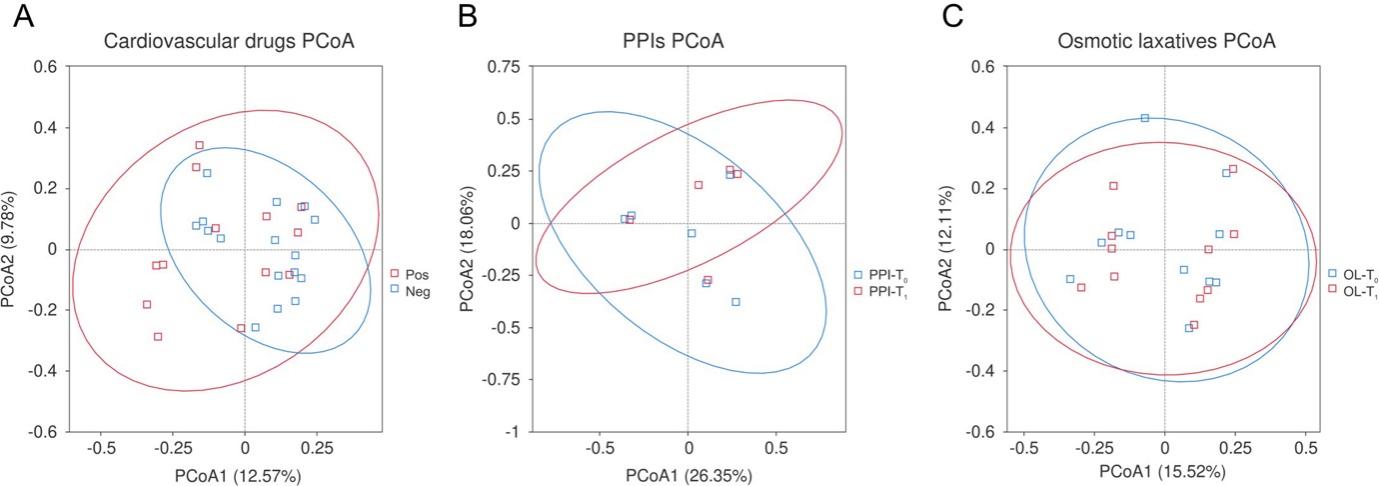
Supplementary Figure 2. Intestinal microbiota of patients with concomitant medications.**

1. Fecal samples collected at baseline from patients with (Pos) or without (Neg) cardiovascular drugs utilization were analyzed. PCoA plot using Bray-Curtis distances is shown. (B) Fecal samples collected before (PPI-T_0_) and after (PPI-T_1_) PPI administration were analyzed. CoA plot using Bray-Curtis distances is shown. (C) Fecal samples collected before (OL-T_0_) and after (OL-T_1_) osmotic laxatives administration were analyzed. CoA plot using Bray-Curtis distances is shown.

Abbreviations: Neg, negative for cardiovascular utilization; OL, osmotic laxatives; OL-T_0_, before osmotic laxatives adminidtration; OL-T_1_, after osmotic laxatives administration; PCoA, principal coordinates analysis; Pos, positive for cardiovascular drug utilization; PPI, proton pump inhibitor; PPI-T_0_, before PPI administration; PPI-T_1_, after PPI administration.

**
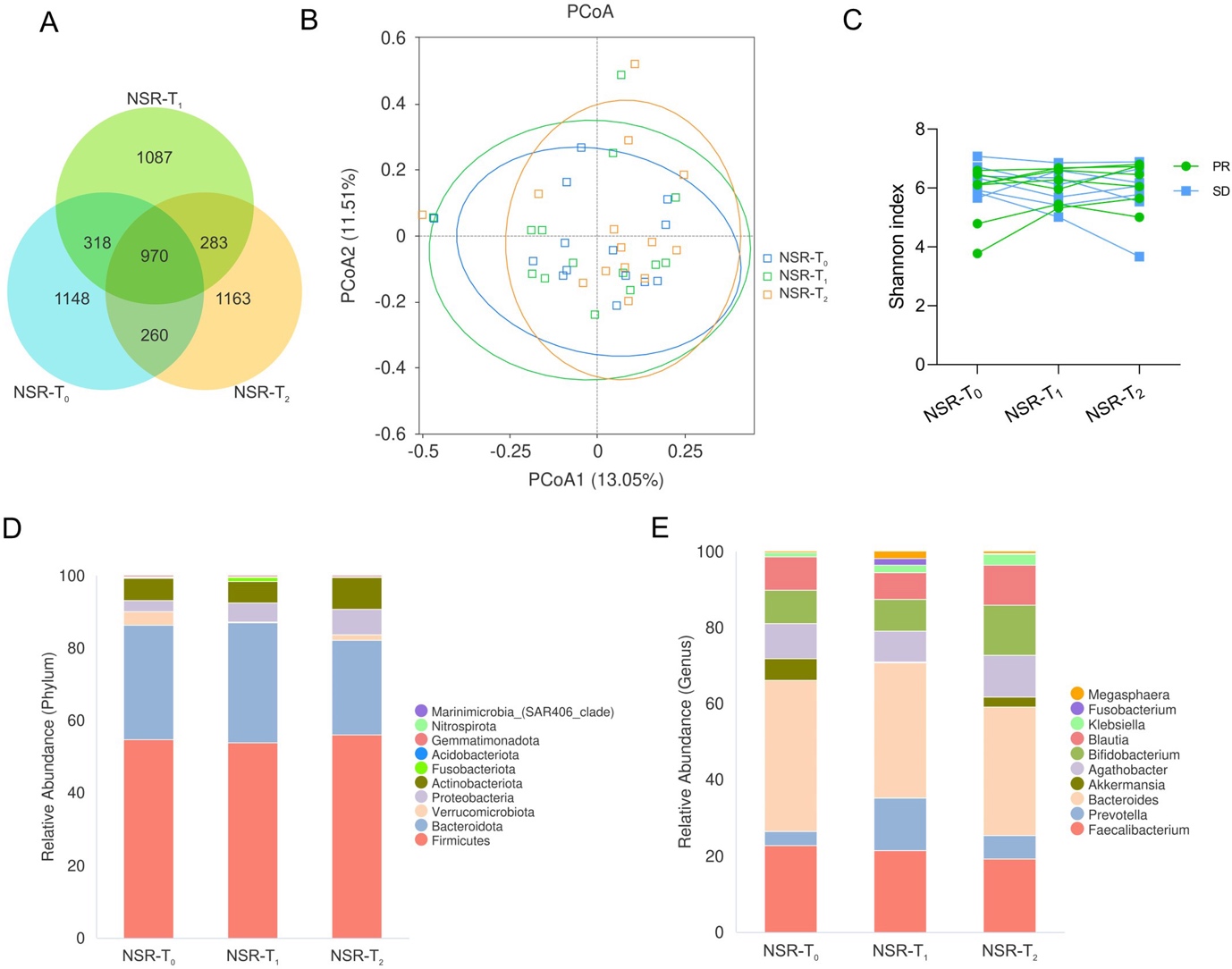
Supplementary Figure 3. The longitudinal intestinal microbiota of patients without secondary resistance to ICI treatment.**

Fecal samples were sequentially collected from baseline (NSR-T_0_) to primary response (NSR-T_1_) to durable response (NSR-T_2_) in patients without secondary resistance. (A) Venn diagram of ASVs; (B) PCoA plot using Bray-Curtis distances; (C) Individual α-diversity by Shannon index; (D) Histogram of intestinal microbiota with top 10 phyla; (E) Histogram of intestinal microbiota with top 10 genera.

Abbreviations: ASVs, amplicon sequence variants; NSR-T_0_, non-secondary resistance patients - before ICI treatment; NSR-T_1_, non-secondary resistance patients - primary response after ICI treatment; NSR-T_2_, non-secondary resistance patients - durable response after ICI treatment; PCoA, principal coordinates analysis; PR, partial response; SD, stable disease.

**
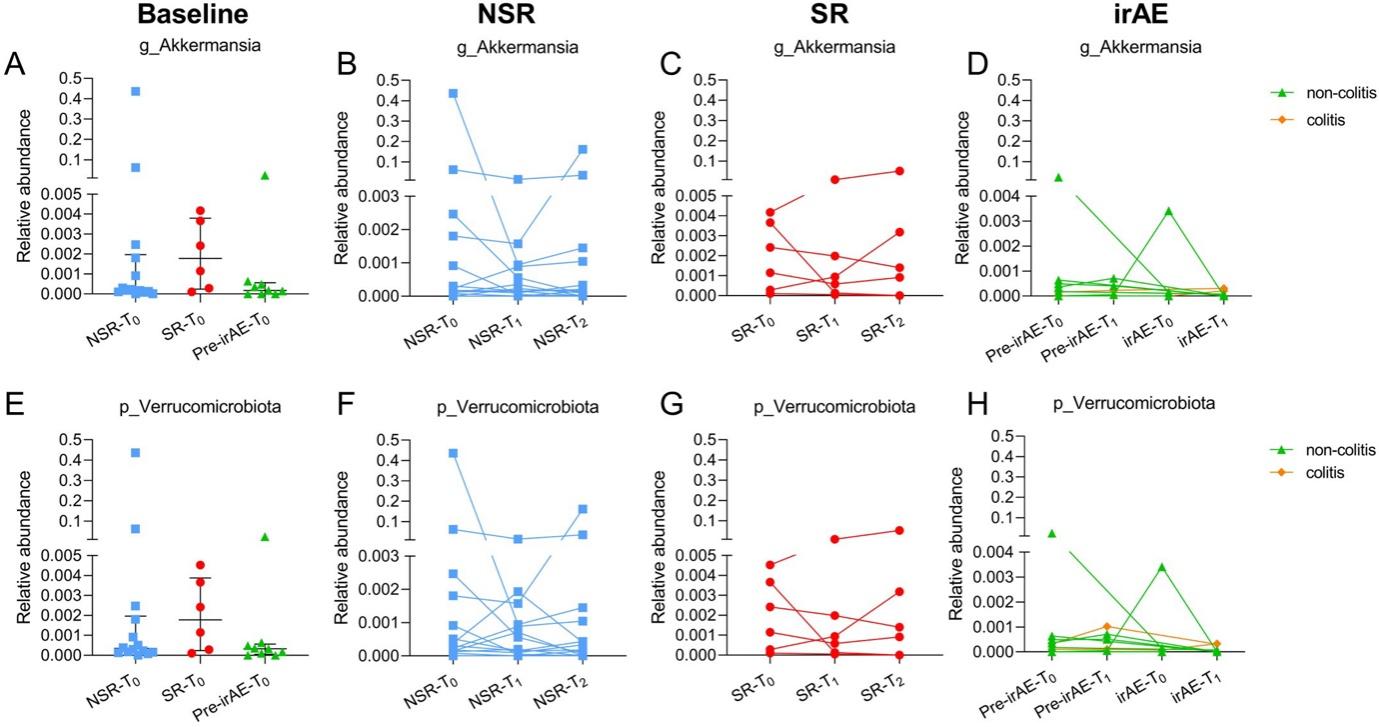
Supplementary Figure 4. Relative Abundance of *Verrucomicrobiota* and *Akkermansia*.**

The relative abundance of *Akkermansia* genus at baseline (A) and longitudinal dynamics in NSR (B), SR (C) and irAE (D) cohorts. The relative abundance of *Verrucomicrobiota* phylum at baseline (E) and longitudinal dynamics in NSR (F), SR (G) and irAE (H) cohorts.

Abbreviations: irAE, immune-related adverse event; irAE-T_0_, irAE patients - after irAE occurrence; irAE-T_1_, irAE patients - after irAE remission; NSR, non-secondary resistance; NSR-T_0_, non-secondary resistance patients - before ICI treatment; NSR-T_1_, non-secondary resistance patients - primary response after ICI treatment; NSR-T_2_, non-secondary resistance patients - durable response after ICI treatment; Pre-irAE-T_0_, irAE patients - baseline before ICI treatment; Pre-irAE-T_1_, irAE patients - after ICI treatment without irAE occurrence; SR, secondary resistance; SR-T_0_, secondary resistance patients - before ICI treatment; SR-T_1_, secondary resistance patients - primary response after ICI treatment; SR-T_2_, secondary resistance patients - secondary resistance after ICI treatment.

**
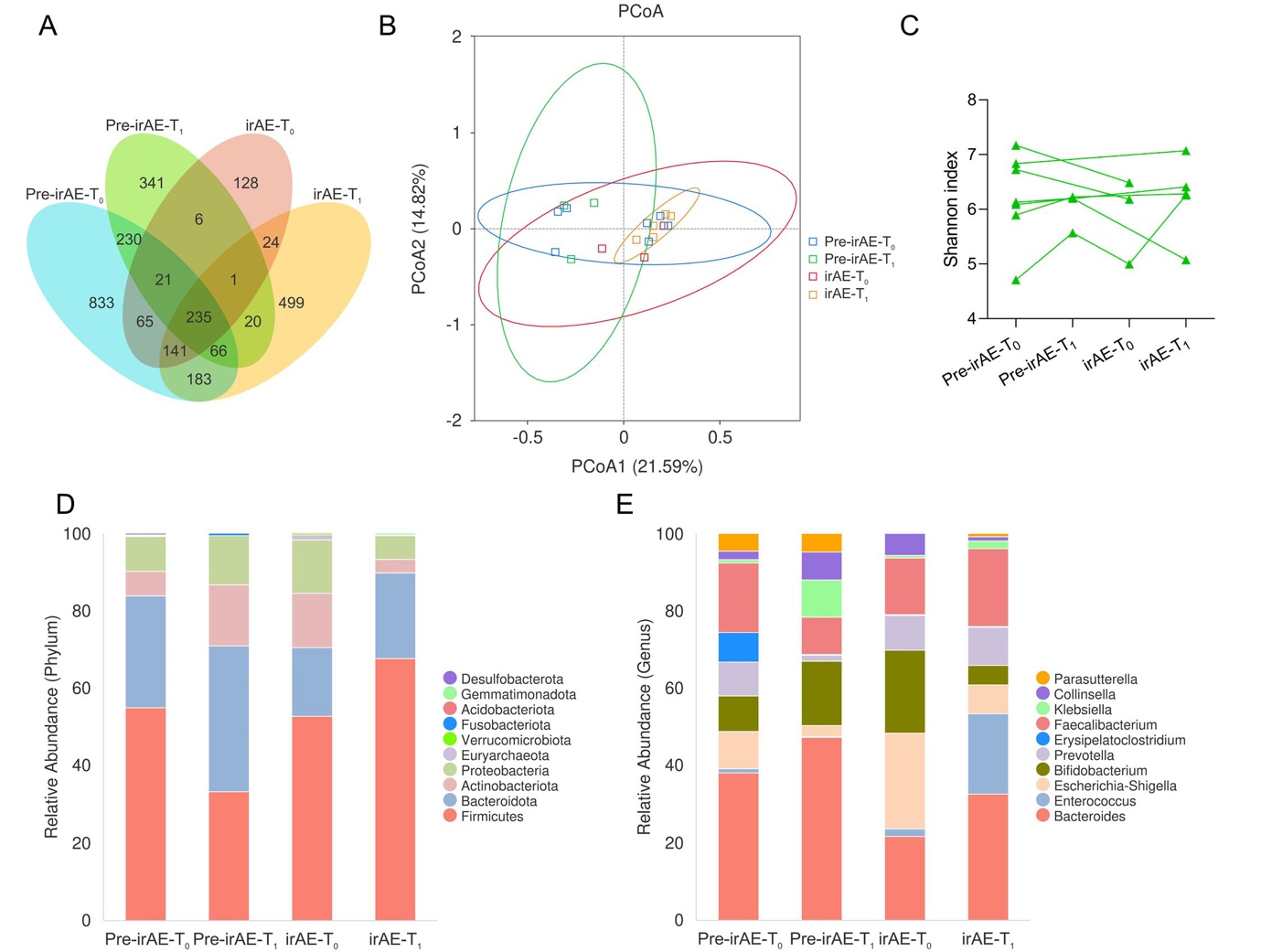
Supplementary Figure 5. The longitudinal intestinal microbiota in patients with non-colitic irAEs.**

Fecal samples were sequentially collected from baseline Pre-irAE-T_0_ to Pre-irAE-T_1_ after ICI treatment without irAEs, irAE-T_0_ with irAE occurrence, and irAE-T_1_ with irAE remission in patients with non-colitic irAEs. (A) Venn diagram of ASVs; (B) PCoA plot using Bray-Curtis distances; (C) Individual α-diversity by Shannon index; (D) Histogram of intestinal microbiota with top 10 phyla; (E) Histogram of intestinal microbiota with top 10 genera.

Abbreviations: irAE, immune-related adverse event; ASVs, amplicon sequence variants; irAE-T_0_, irAE patients - after non-colitic irAE occurrence; irAE-T_1_, irAE patients - after non-colitic irAE remission; PCoA, principal coordinates analysis; Pre-irAE-T_0_, irAE patients - baseline before ICI treatment; Pre-irAE-T_1_, irAE patients - after ICIs treatment without irAE occurrence.

**Supplementary Table 1 Medication regimens**

| Medication regimens | Patients with secondary resistance (SR) (N=6) | Patients without secondary resistance (NSR) (N=14) | irAE patients  (N=9) | *P*-value |
| --- | --- | --- | --- | --- |
| ICIs |  |  |  | 0.466 |
| anti-PD-1 | 5 (93.3%) | 13 (92.9%) | 9 (100.0%) |  |
| anti-PD-L1 | 1 (16.7%) | 1 (7.1%) | 0 (0.0%) |  |
| Concomitant chemotherapy |  |  |  | 0.833 |
| Pemetrexed + Carboplatin | 3 (50.0%) | 7 (50.0%) | 5 (55.5%) |  |
| Paclitaxel + Carboplatin | 2 (33.3%) | 5 (35.7%) | 3 (33.3%) |  |
| Others | 1 (16.7%) | 2 (14.3%) | 0 (0.0%) |  |
| No concomitant chemotherapy | 0 (0.0%) | 0 (0.0%) | 1 (11.1%) |  |
| Antiemetics | 6 (100.0%) | 14 (100.0%) | 9 (100.0%) | - |
| Cardiovascular drugs | 1 (16.7%) | 6 (42.9%) | 6 (66.7%) | 0.162 |
| Proton pump inhibitors | 2 (33.3%) | 2 (14.3%) | 2 (22.2%) | 0.721 |
| Osmotic laxatives | 2 (33.3%) | 7 (50.0%) | 4 (44.4%) | 0.888 |

anti-PD-1, anti-programmed death-1, including Pembrolizumab, Toripalimab, Sintilimab, Tislelizumab or Camrelizumab; anti-PD-L1, anti-programmed death ligand 1, including Durvalumab or Atezolizumab.

**Supplementary Table 2 Alpha diversity and bacterial relative abundance**

| Group | Shannon index (mean±SD) | Relative abundance of *Lachnospiraceae* (median - IQR) | Relative abundance of *Ruminococcaceae* (median - IQR) | Relative abundance of *Agathobacter* (median - IQR) | Relative abundance of *Faecalibacterium* (median - IQR) | Relative abundance of butyrate-producing bacteria (median - IQR) | Relative abundance of IgA-coated bacteria (median - IQR) | Relative abundance of *Bacteroides* (median - IQR) | Relative abundance of *Verrucomicrobiota* (median - IQR) | Relative abundance of *Akkermansia* (median - IQR) |
| --- | --- | --- | --- | --- | --- | --- | --- | --- | --- | --- |
| SR-T_0_ | 6.04 (5.45-6.64) | 0.29 (0.26-0.30) | 0.16 (0.15-0.20) | 0.03 (0.02-0.10) | 0.12 (0.08-0.17) | 0.25 (0.24-0.29) | 0.05 (0.04-0.09) | 0.22 (0.14-0.30) | 0.002 (0.000-0.003) | 0.002 (0.000-0.003) |
| SR-T_1_ | 6.22 (5.51-6.93) | 0.30 (0.23-0.35) | 0.16 (0.13-0.19) | 0.05 (0.02-0.07) | 0.12 (0.08-0.17) | 0.26 (0.25-0.32) | 0.07 (0.06-0.08) | 0.17 (0.13-0.24) | 0.001 (0.000-0.002) | 0.001 (0.000-0.002) |
| SR-T_2_ | 6.12 (5.45-6.78) | 0.20 (0.14-0.31) | 0.13 (0.09-0.14) | 0.02 (0.01-0.03) | 0.08 (0.05-0.10) | 0.20 (0.16-0.22) | 0.07 (0.04-0.09) | 0.14 (0.12-0.28) | 0.001 (0.000-0.003) | 0.001 (0.000-0.003) |
| NSR-T_0_ | 6.00 (5.16-6.83) | 0.23 (0.17-0.40) | 0.15 (0.12-0.22) | 0.04 (0.02-0.06) | 0.11 (0.08-0.15) | 0.24 (0.17-0.32) | 0.08 (0.05-0.13) | 0.22 (0.16-0.30) | 0.000 (0.000-0.002) | 0.000 (0.000-0.002) |
| NSR-T_1_ | 6.07 (5.47-6.67) | 0.27 (0.16-0.38) | 0.14 (0.11-0.21) | 0.03 (0.02-0.09) | 0.10 (0.08-0.19) | 0.28 (0.23-0.33) | 0.06 (0.04-0.10) | 0.22 (0.11-0.30) | 0.000 (0.000-0.001) | 0.000 (0.000-0.001) |
| NSR-T_2_ | 6.02 (5.14-6.89) | 0.37 (0.16-0.39) | 0.16 (0.14-0.21) | 0.04 (0.02-0.10) | 0.11 (0.07-0.13) | 0.25 (0.20-0.27) | 0.11 (0.07-0.14) | 0.20 (0.16-0.22) | 0.000 (0.000-0.001) | 0.000 (0.000-0.001) |
| Pre-irAE-T_0_ | 6.25 (5.53-6.96) | - | - | - | - | 0.26 (0.20-0.37) | 0.06 (0.03-0.07) | 0.12 (0.10-0.18) | 0.000 (0.000-0.000) | 0.000 (0.000-0.000) |
| Pre-irAE-T_1_ | 6.09 (5.54-6.64) | - | - | - | - | 0.21 (0.19-0.21) | 0.03 (0.03-0.22) | 0.10 (0.07-0.36) | 0.001 (0.000-0.001) | 0.000 (0.000-0.000) |
| irAE-T_0_ | 5.80 (5.13-6.46) | - | - | - | - | 0.16 (0.13-0.22) | 0.13 (0.10-0.16) | 0.08 (0.06-0.12) | 0.000 (0.000-0.001) | 0.000 (0.000-0.001) |
| irAE-T_1_ | 6.16 (5.53-6.78) | - | - | - | - | 0.23 (0.19-0.25) | 0.05 (0.04-0.05) | 0.10 (0.09-0.18) | 0.000 (0.000-0.000) | 0.000 (0.000-0.000) |
| Pre-irAE-T_0_ (non-colitis) | 6.22 (5.41-7.03) | - | - | - | - | 0.23 (0.18-0.27) | 0.06 (0.02-0.08) | 0.12 (0.12-0.28) | - | - |
| Pre-irAE-T_1_ (non-colitis) | 6.00 (5.62-6.37) | - | - | - | - | 0.20 (0.19-0.21) | 0.03 (0.02-0.18) | 0.36 (0.23-0.41) | - | - |
| irAE-T_0_ (non-colitis) | 5.89 (5.10-6.67) | - | - | - | - | 0.18 (0.15-0.25) | 0.17 (0.09-0.19) | 0.09 (0.07-0.14) | - | - |
| irAE-T_1_ (non-colitis) | 6.22 (5.50-6.94) | - | - | - | - | 0.23 (0.21-0.24) | 0.05 (0.04-0.05) | 0.16 (0.10-0.21) | - | - |

Abbreviations: IQR, interquartile; irAE, immune-related adverse event; irAE-T_0_, irAE patients - after irAE occurrence; irAE-T_1_, irAE patients - after irAE remission; NSR, non-secondary resistance; NSR-T0, non-secondary resistance patients - before ICI treatment; NSR-T_1_, non-secondary resistance patients - primary response after ICI treatment; NSR-T_2_, non-secondary resistance patients - durable response after ICI treatment; Pre-irAE-T_0_, irAE patients - baseline before ICI treatment; Pre-irAE-T_1_, irAE patients - after ICI treatment without irAE occurrence; SD, standard deviation; SR, secondary resistance; SR-T_0_, secondary resistance patients - before ICI treatment; SR-T_1_, secondary resistance patients - primary response after ICI treatment; SR-T_2_, secondary resistance patients - secondary resistance after ICI treatment.
